# Correction to: A preclinical model to investigate normal tissue damage following fractionated radiotherapy to the head and neck

**DOI:** 10.1093/jrr/rrad061

**Published:** 2023-09-05

This is a correction to: Inga Solgård Juvkam, Olga Zlygosteva, Delmon Arous, Hilde Kanli Galtung, Eirik Malinen, Tine Merete Søland, Nina Jeppesen Edin, A preclinical model to investigate normal tissue damage following fractionated radiotherapy to the head and neck, *Journal of Radiation Research*, Volume 64, Issue 1, January 2023, Pages 44–52, https://doi.org/10.1093/jrr/rrac066

In this work, we used a lead collimator to limit the radiation field to certain parts of the mouse head, as described in the original manuscript. The original dosimetry was performed without the lead shield, as we were concerned that the resulting collimation would lead to partial volume effects, and thus systematic errors, in the ion chamber measurements. However, the impact of the lead plate on the dose to the irradiated area was underestimated. This was evident from additional dosimetry of the irradiation setup with and without the plate performed after the original work was published. This revised dosimetry is given below.

The absorbed dose rate to water of the X-ray system was measured to be 0.63 ± 0.02 Gy/min for an unshielded beam using a FC65-G Farmer type ionization chamber (IBA Dosimetry, Germany) together with a MAX-4000 electrometer (Standard Imaging, USA) according to IAEA report TRS-277 [1-2]. To measure the dose in the collimated case, we employed Gafchromic*™* EBT3 dosimeter films at a source-surface distance representing sagittal skin entrance of the mouse. Calibration was done using an unshielded beam. EBT3 films (lot No. 02122001) were irradiated and processed according to the recommended protocols specified for radiochromic film dosimetry in the report of AAPM Task Group 235 [3].

The results showed that the actual dose with the use of the collimator lead plate was 12% lower than the dose reported in the original manuscript. Therefore, all the doses in the manuscript should be corrected accordingly: 3 Gy to 2.6 Gy, 4.4 Gy to 3.9 Gy, 5 Gy to 4.4 Gy, 5.75 Gy to 5.1 Gy, 6.5 Gy to 5.7 Gy, 7.5 Gy to 6.6 Gy, 8.5 Gy to 7.5 Gy.

The authors apologize for any inconvenience this might have caused.

[1]: IAEA, Report No. ISBN 92-0-115 087-3, 1987.

[2]: IAEA, Report No. ISSN 1011-4289, 1996.

[3]: Niroomand-Rad A, Chiu-Tsao S-T, **Grams** M P, Lewis D F, Soares C G, Van Battum L J, Das I J, Trichter S, Kissick M W and Massillon-Jl G 2020 Report of AAPM task group 235 radiochromic film dosimetry: an update to TG-55 *Med. Phys*  **47**5 986-6025.

**NB**: the error in dosimetry is emended in this correction notice alone. The errors have not been emended throughout the paper online.

